# An Electromechanical Lab-on-a-Chip Platform for Colorimetric Detection of Serum Creatinine

**DOI:** 10.1021/acsomega.2c03354

**Published:** 2022-07-15

**Authors:** Betul Karakuzu, Ergun Alperay Tarim, Cemre Oksuz, H. Cumhur Tekin

**Affiliations:** † Department of Bioengineering, Izmir Institute of Technology, Izmir 35430, Turkey; ‡ METU MEMS Center, Ankara 06520, Turkey

## Abstract

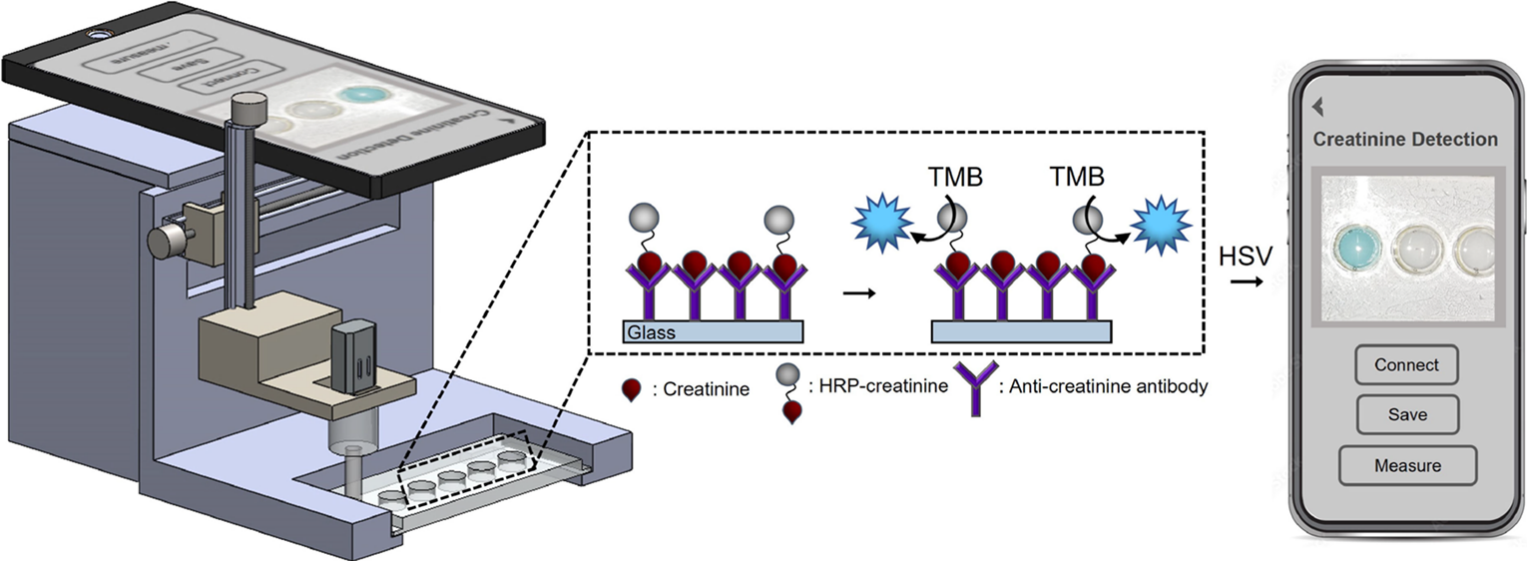

Chronic kidney disease (CKD) is a high-cost disease that affects approximately one in ten people globally, progresses rapidly, results in kidney failure or dialysis, and triggers other diseases. Although clinically used serum creatinine tests are used to evaluate kidney functions, these tests are not suitable for frequent and regular control at-home settings that obstruct the regular monitoring of kidney functions, improving CKD management with early intervention. This study introduced a new electromechanical lab-on-a-chip platform for point-of-care detection of serum creatinine levels using colorimetric enzyme-linked immunosorbent assay (ELISA). The platform was composed of a chip containing microreservoirs, a stirring bar coated with creatinine-specific antibodies, and a phone to detect color generated via ELISA protocols to evaluate creatinine levels. An electromechanical system was used to move the stirring bar to different microreservoirs and stir it inside them to capture and detect serum creatinine in the sample. The presented platform allowed automated analysis of creatinine in ∼50 min down to ∼1 and ∼2 mg/dL in phosphate-buffered saline (PBS) and fetal bovine serum (FBS), respectively. Phone camera measurements in hue, saturation, value (HSV) space showed sensitive analysis compared to a benchtop spectrophotometer that could allow low-cost analysis at point-of-care.

## Introduction

Chronic kidney disease (CKD) is a disease that progresses rapidly, is irreversible, and brings many complications.^1,2^ If the disease progression is not monitored regularly, it can rapidly worsen and result in end-stage renal disease with kidney failure, which needs dialysis or renal replacement therapy.^3^ Because of this, CKD needs quick screening and regular monitoring.^4^

Glomerular filtration rate (GFR) is used to evaluate CKD stages by measuring renal clearance with endogenous or exogenous substances.^5^ Instead of using this complex method, estimated GFR can be calculated from the serum creatinine level using different formulas with age, race, and gender information.^6,7^ For this purpose, the serum creatinine level can be determined using the Jaffe method, enzymatic methods, isotope dilute gas chromatography–mass spectrometry (IDGC-MS), high-performance liquid chromatography (HPLC), Fourier-transform infrared spectroscopy (FTIR), and thin-layer chromatography (TLC).^8−13^ However, these methods’ cost and lengthy analysis time limit their usage at point-of-care.

Serum creatinine levels ranging 0–11.3 mg/dL (0–1000 μM), where 0.6–1.2 mg/dL for men and 0.5–1.1 mg/dL for women are normal results in healthy patients,^14^ can also be detected using different biosensors. For instance, creatinine can be detected in a concentration range of 0–11.33 mg/dL using a conductive polymer-based biosensor.^15^ Electrochemical measurements were used to detect the binding of horseradish peroxidase (HRP)-conjugated creatinine antibody with creatinine in the presence of tetramethylbenzidine (TMB). Furthermore, a creatinine biosensor based on the capacitive detection of creatinine antibodies bound to the immobilized creatinine layer in the presence of serum creatinine allowed the detection of serum creatinine in the range of 0–10 mg/dL.^16^ Creatinine measurement was also conducted on the modified carbon paste electrode down to 0.008 μM levels.^17^ Enzymatic hydrolysis created a redox signal in a developed electrochemical biosensor.^18^ In this biosensor, the measurement was conducted in the 0.24–4 mg/dL creatinine concentration range with 300 μL sample. A dual electrochemical sensor on a test strip was also utilized for creatinine measurement in the presence of hydrogen peroxide.^19^ When the sample flows through the device, hydrogen peroxide is produced with the reaction of three enzymes in the immobilization area. Moreover, creatinine can be detected using a molecularly imprinted polymer.^20^ However, these methods require many production steps, increasing the cost and device complexity of biosensors that limit their usage. On the other hand, lab-on-a-chip (LOC) systems are good candidates to perform creatinine detection at point-of-care by automating complex assay protocols, including preparation, processing, and analyzing samples.^21−23^ For example, creatinine detection from whole blood was achieved on a three-dimensional (3D) paper-based microfluidic device.^24^ Separated serum in this device was analyzed using the Jaffe method. The obtained color through the Jaffe reaction was evaluated with a CMOS camera using intensity values of green and blue channels that allowed detection of serum creatinine in the range of 0.19–7.64 mg/dL. Furthermore, creatinine can be measured using surface plasmon resonance on a polydimethylsiloxane (PDMS) microfluidic device.^25^ Au film on this device was coated with HRP-linked osmium-poly (vinyl pyridine) and creatinine-specific enzyme. Creasensor including the SIMPLE-based biosensor and the microfluidic cartridge was used for colorimetric creatinine measurement with a range of 0.76–20 mg/dL.^26^ However, these devices required manual pumps, integrated heated modules, and contained expensive device components for complete creatinine analysis.

LOC devices can combine fluidics, electronics, and optics on a single device for standalone operation.^27,28^ Automated fluidic operations on these devices require valves, mixers, and pumps that can create difficulties in integrating and controlling all these elements realized with a complex fabrication process.^29,30^ Electromechanical LOC devices can provide self-driving operations with electronic and mechanical components for fully automated assay protocols.^31^ For instance, automatic water analysis was achieved with a 3D-printed robotic system coupled with a syringe pump to transfer samples and reagents, temperature and conductivity sensors to provide accurate measurement, and a webcam for colorimetric pH analysis on a 96-well plate.^32^ Moreover, a robotic system was used to perform automated ELISA protocols for chloramphenicol detection using colorimetric measurements.^33^ Protocols were conducted on a microfluidic chip, where pneumatically driven loading, mixing, and washing steps were achieved with a robotic arm. A fully automated microfluidic platform was also developed for CD4 cell counting with colorimetric ELISA protocols.^34^ In this platform, magnetic beads used as assay surfaces were transferred between different chambers separated by mineral oils using a motorized magnet stage. Different assay protocols from purification to detection of CD4 cells were conducted in each chamber. These electromechanical LOC systems can be very convenient in routine clinical and point-of-care testing. With 3D printing, these systems can be fabricated rapidly in a cost-effective way. They can also enable plug-and-play and standalone operations with no or minimal preparation, which can significantly benefit LOC systems’ usability.

This study presents a new electromechanical LOC system that enables the automated detection of creatinine. The electromechanical system allows the movement and stirring of a creatinine-specific antibody-coated bar on a chip containing microreservoirs. These microreservoirs are filled with necessary ELISA solutions to detect creatinine in a drop of sample, and the analysis is conducted in a plug-and-play format. After the automated assay protocols, the creatinine is detected using colorimetric analysis with a simple smartphone camera placed on the platform. The captured images are analyzed in HSV space to evaluate creatinine levels. By doing so, the electromechanical LOC system allows for the detection of creatinine down to 1 mg/dL in 180 μL of phosphate-buffered saline (PBS) and 2 mg/dL in 18 μL of fetal bovine serum (FBS). The low-cost and portable platform offers automated creatinine detection in ∼50 min. This practical measurement method shows similar performance compared to the benchtop spectrophotometer.

## Experimental Section

### Electromechanical LOC Platform

**Figure 1:**
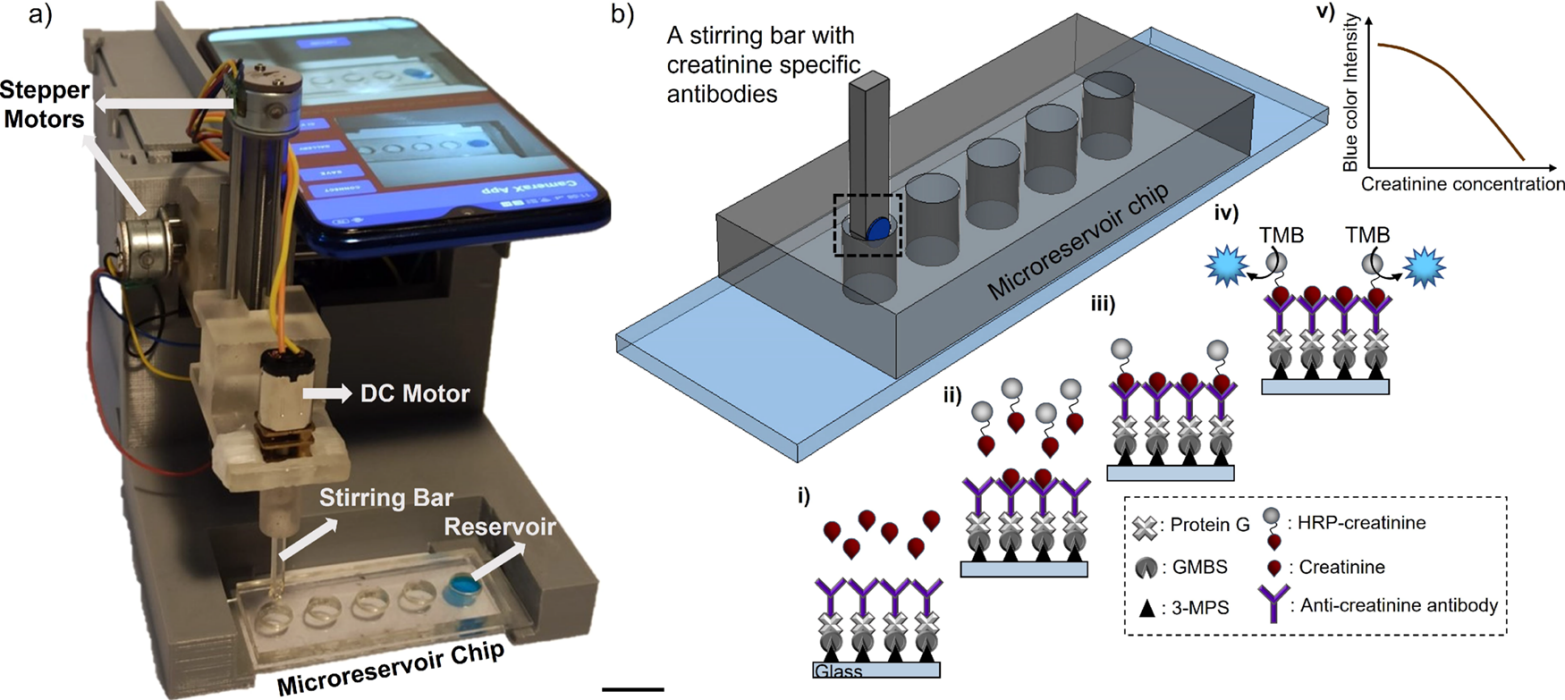
An electromechanical lab-on-a-chip platform for automated creatinine analysis. (a) Photograph of the platform. Scale bar is 2 cm. (b) Creatinine detection protocol. (i) An antibody-coated stirring bar enters the sample in the first microreservoir, and the mixing process is started by rotating the stirring bar. At this stage, the creatinine in the sample is captured by the antibodies on the stirring bar. (ii) Stirring bar then enters the second microreservoir containing the HRP-creatinine and is mixed there. The antibodies capture HRP-creatinine. (iii) Stirring bar is then washed in the washing solution in the third and fourth microreservoirs. (iv) Then, the stirring bar is passed into the last microreservoir containing the TMB and mixed. TMB reacts enzymatically with HRP, and (v) a blue color appears, which is inversely proportional to the creatinine concentration in the sample.

Our platform contained an electromechanical system that automatically conducted the creatinine detection protocol in a microreservoir chip (Supporting Information) by transferring and mixing the stirring bar (Supporting Information) in consecutive microreservoirs. For this purpose, 2 DC stepper motors with linear guide rails (D8-MOTOR80, Tools Shopping Center Store, Shenzhen, China) to transfer the stirring bar to different microreservoirs and one DC motor (Micro Metal Gearmotor HPCB 3061, Pololu Robotics and Electronics, Las Vegas, USA) to conduct mixing inside microreservoirs were used. The frame of the platform was fabricated using the polylactic acid (PLA) filament (Ultimaker, Utrecht, Netherlands) in a 3D printer (Ultimaker 2+, Ultimaker, Utrecht, Netherlands), and the motors were arranged on it (Figure 1a). The platform has a size of 10 cm × 10 cm × 20 cm and a weight of 390 g. The motors were controlled with a microcontroller (Arduino Mega 2560 R3, Arduino LLC, Boston, USA) equipped with a motor-driver board (CNC Expansion Board V3.0, Shenzhen HiLetgo Technology Co. Ltd., Shenzhen, China), two motor drivers (A4988, Pololu Robotics, and Electronics, Las Vegas, USA), DC motor driver circuit, which have Darlington transistor (BDX53C, STMicroelectronics, Switzerland), 100 ohms ±5% resistance, and diode (1 N4007, MIC Electronic Co., Shanghai), and a Bluetooth module (HC-06 Bluetooth Module, Shenzhen HiLetgo Technology Co. Ltd., Shenzhen, China) (Figure S2). Stepper motors were driven with 7 V to reach ∼9 mm/s speed on their carriages to move the stirring bar. The voltage induced on the DC motor could be adjusted between 0 and 15 V to change the rotation speed (Figure S3). The platform can be remotely controlled with an in-house-developed Android application (Figure S4 and Movie 1) through Bluetooth connection to conduct creatinine detection protocols.

### Creatinine Detection Protocol Conducted on the Electromechanical LOC Platform

The microreservoir chip was filled with the creatinine sample on its first microreservoir, 1:400 HRP-creatinine solution on its second microreservoir, 0.05% pluronic solution on its third and fourth microreservoirs, and TMB solution on its last microreservoir. Each microreservoir contained 180 μL of the corresponding solution. A stirring bar with creatinine-specific antibodies on its tip was also mounted on the electromechanical system. To conduct the creatinine detection, the stirring bar was automatically dipped into different microreservoirs (Figure S5 and Movie 1), and the following protocol was followed: (i) stirring 10 min at 1000 rpm in the first and the second microreservoirs, respectively, (ii) stirring 1 min at 1000 rpm in the third and the fourth microreservoirs, respectively, and (iv) stirring 30 min at 3000 rpm in the fifth microreservoir. By doing so, creatinine in the sample was captured on the stirring bar, and then, HRP-creatinine was caught on the remaining antibodies on the stirring bar. After the stirring bar was cleaned twice in the pluronic solution to eliminate unspecific binding, HRP-creatinine captured on the stirring bar was reacted with TMB on the last microreservoir, and blue color appeared. The intensity of the blue color was inversely proportional to the captured creatinine from the sample solution (Figure 1b). The appeared color was analyzed with a benchtop spectrophotometer using absorbance measurements at 650 nm (Multiscan Go, Thermo Fisher Scientific, Massachusetts, USA) and a smartphone camera (OPPO RX17 Neo, OPPO Electronics Corp., Dongguan, China). Images were taken at 10 cm from the top of the platform in an unlit environment with a flash of a smartphone. A closed box, which eliminates the effect of ambient light changes on analysis, is used to cover the electromechanical LOC system during imaging. The images taken by focusing with the fixed focus feature of the mobile phone were saved for analysis in RGB and HSV color spaces. The mean intensity values of red, green, and blue channels were used for the detection. For the detection with HSV color space, hue (0.194< and 0.612>), saturation (0.013< and 1.0>), and brightness (0.0< and 0.933>) parameters were fixed for each image. Thanks to the HSV color space features, the camera, ambient light, and photograph resolution affect the analysis at a minimum level.^35^ The recorded images were analyzed using the MATLAB program to find the color intensities in RGB and HSV color spaces.

For the optimization of protocol parameters, antibody, and protein-G concentration, mixing time for HRP and TMB microreservoirs, mixing speed, and the ratio of HRP-creatinine were analyzed. The creatinine sample was prepared in 180 μL of PBS or 18 μL of FBS. FBS samples were further diluted with 162 μL of PBS in the first microreservoir.

### Statistical Analysis

Data were shown as the mean ± standard deviation (SD) of at least 3 replicates of experiments. For statistical analysis, an unpaired Student’s t-test was conducted on the data. The statistical significance threshold was set to 0.05 (*p* < 0.05). The coefficient of determination (*R*^2^) values of linear regression models were presented in the figures. These analyses were performed using GraphPad software (Prism 8 version, GraphPad, USA).

## Results and Discussion

In the developed electromechanical LOC platform (Figure 1), an automated creatinine detection protocol was conducted in the microreservoir chip. The protocol contained transferring the stirring bar consequently between different microreservoirs filled with different assay solutions and mixing inside these microreservoirs by rotating the stirring bar to enhance and fasten the analysis.

### Surface Functionalization of the Glass Substrate

The success of the surface functionalization protocols was evaluated with FTIR analysis (Figure S6, Supporting Information). As a result, it was concluded that the glass surface was successfully functionalized with anti-creatinine antibodies.

The concentration of protein-G and anti-creatinine antibodies used for surface functionalization protocols was also studied to determine the concentration to saturate the glass surface. As the surface becomes saturated with anti-creatinine antibodies, it is expected that less-fluorescent IgG will be captured on the surface, and the fluorescent signal on the surface will decrease (Figures S7 and S8). Moreover, the effect of antibody concentration on creatinine detection was examined. Equal absorbance signals were observed for 10 and 100 μg/mL antibodies while detecting 0 μg/mL of creatinine (Figure S9). Because of that, 10 μg/mL creatinine antibody and 100 μg/mL Protein-G were used in the platform.

### Mixing Performance of the Electromechanical LOC Platform

**Figure 2:**
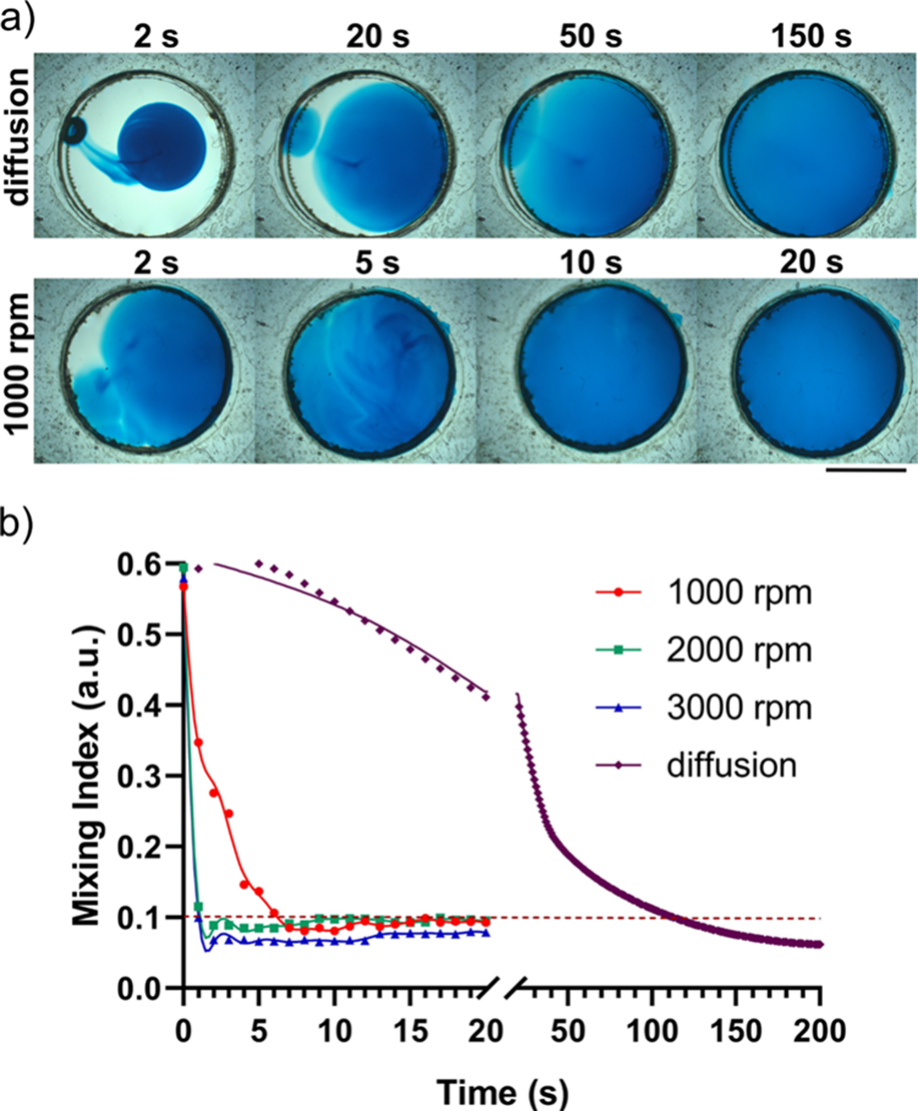
Effect of the stirring bar’s rotation speeds on mixing inside the microreservoir. (a) Micrographs of microreservoirs were taken with mixing at 1000 rpm and pure diffusion-based mixing. The scale bar is 4 mm. (b) Mixing index values for mixing at different speeds (1000–3000 rpm) and pure diffusion-based mixing.

On the platform, the stirring bar was rotated with a DC motor to enhance mixing within the microreservoirs. The effect of rotation speeds was evaluated on the mixing performance (Figure 2). The mixing index value (eq 1 in Supporting Information)^36^ was decreased below 0.1, which indicates an adequate mixing profile,^37^ in <7 s for different rotation speeds (1000–3000 rpm). On the other hand, adequate mixing was achieved only in ∼110 s with pure diffusion-based mixing, which was at least 16-fold slower than active mixing with rotation. As rotation speed was increased, a very rapid mixing profile was observed. However, a high rotation speed (>3000 rpm) could splash the solutions from microreservoirs that could create unwanted contamination.^38^ Furthermore, a high rotation speed (>3000 rpm) could also detach the glass substrate from the stirring bar, which can interrupt assay protocols. Because of these reasons, the stirring bar was rotated at ≤3000 rpm in the assay protocol.

### Optimization of Electromechanical Protocol Parameters for Creatinine Detection

**Figure 3:**
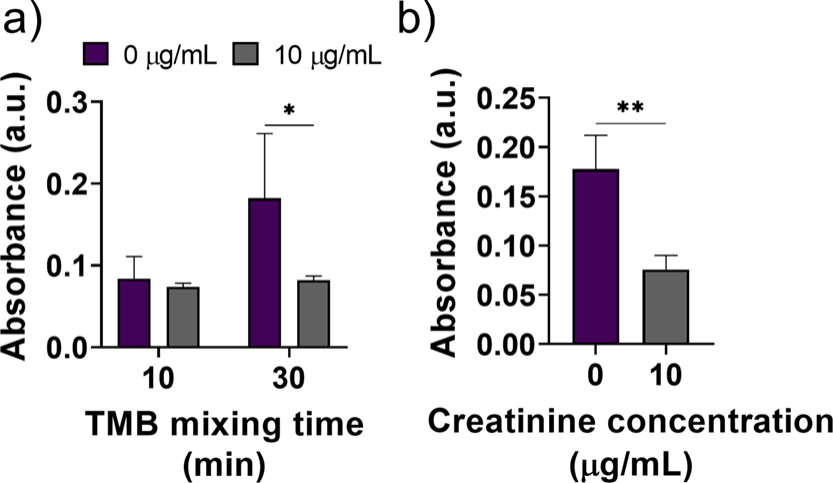
Effect of electromechanical protocol parameters on creatinine detection. (a) Absorbance measurements conducted using a detection protocol with 0 or 10 μg/mL creatinine samples for different mixing times at TMB microreservoirs. 3000 rpm mixing was used in all protocol steps. (b) Absorbance graph obtained for the creatinine detection protocol conducted with 1000 rpm mixing at first four microreservoirs and 30 min mixing at 3000 rpm at TMB microreservoir. (*) and (**) indicate *p* < 0.05 and *p* < 0.01, respectively.

A high-speed mixing by rotating stirring bar at 3000 rpm in microreservoirs was utilized for fast analysis, and the creatinine detection protocol was conducted. Because the most critical step for creatinine detection was the enzymatic reaction of HRP and TMB, different mixing times at the TMB microreservoir were evaluated. While mixing time was increased from 10 to 30 min, statistical differences between the 0 and 10 μg/mL creatinine were observed (Figure 3a). Hence, TMB mixing time was set to 30 min in the protocol.

However, a high-speed mixing profile can induce shear stress that can detach absorbed molecules from the stirring bar in the first four microreservoirs and can alter the detection signal. For this purpose, the mixing speed was reduced to 1000 rpm on the first four microreservoirs. We observed that error bars on the measurement signals were reduced, and the statistical difference between 0 and 10 μg/mL creatinine was increased (Figure 3b). For this reason, 1000 rpm mixing was chosen to be utilized in the first four microreservoirs, while 3000 rpm mixing was set for the TMB microreservoir.

### Creatinine Detection

**Figure 4:**
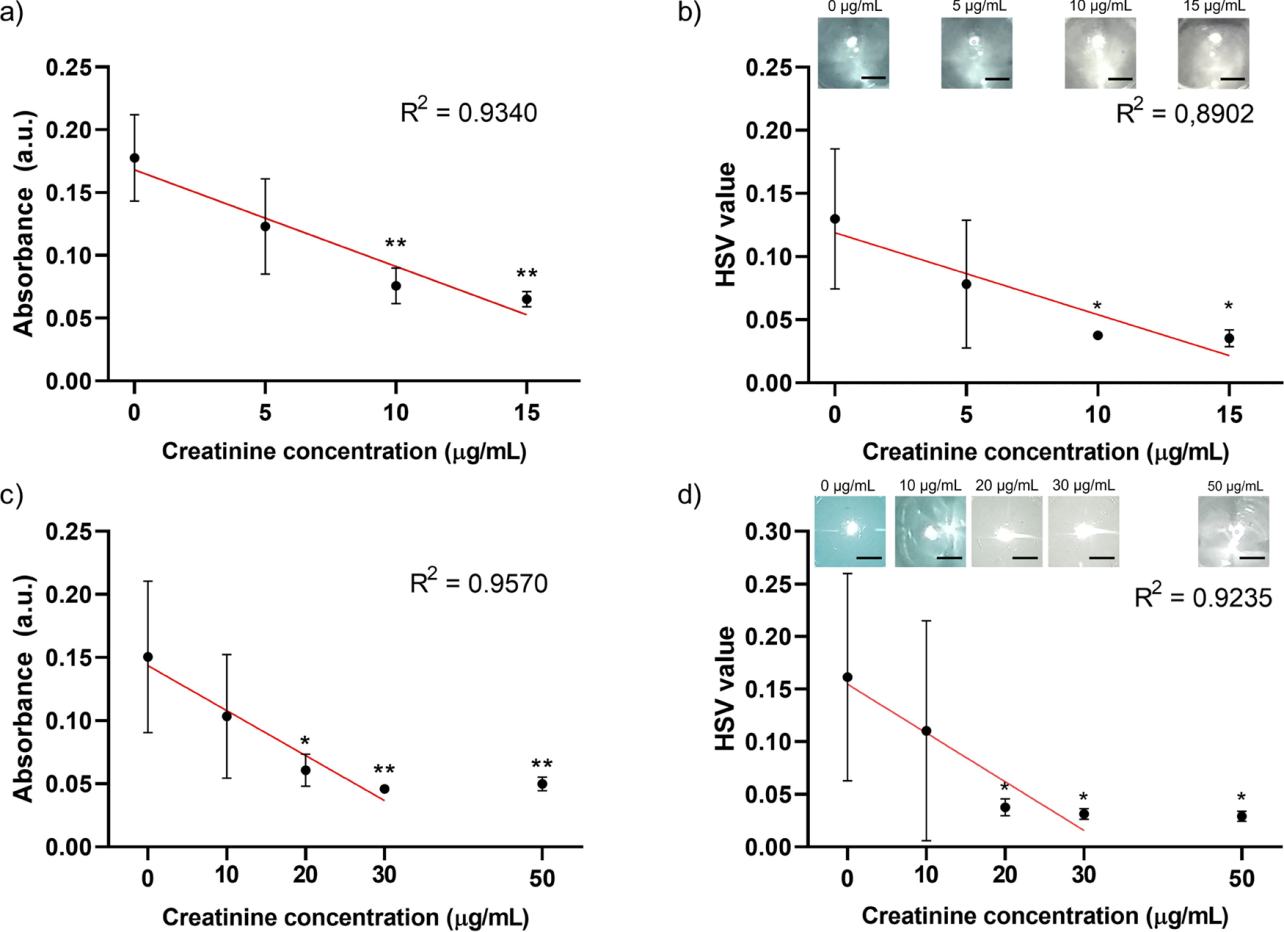
Absorbance values and mean intensity values in HSV spaces for creatinine detection in (a, b) PBS and (c, d) FBS, respectively. Statistical differences according to 0 μg/mL creatinine measurements were indicated on the graphs. Images of the last microreservoirs in creatinine experiments were also given in the figures. (*) and (**) indicate *p* < 0.05 and *p* < 0.01, respectively. The scale bars are 2 mm.

Creatinine in the concentration range of 0–15 μg/mL prepared in PBS was analyzed on the electromechanical LOC platform. As shown in Figure 4a, absorbance levels measured at the TMB microreservoir decreased as the amount of creatinine increased, and a statistical difference was observed for ≥10 μg/mL (i.e., ≥1 mg/dL) concentration levels compared to the control group without creatinine (0 μg/mL). The obtained color in the TMB microreservoir was also analyzed using a smartphone camera. Low correlation and statistical difference with creatinine concentration were obtained using the mean intensity of captured image’s red/blue/green channels (Figure S10). On the other hand, similar correlation values and statistical differences were achieved using the mean intensity values at the HSV space (Figures 4b and S11). The primary color range can be determined sharper than RGB channels with the hue feature in HSV color space.^39^ Besides, analysis closer to absorbance values can be obtained by focusing on the saturation of the primary color. These features provide better measurements than RGB channels.^40^ Creatinine detection was also conducted in FBS with the creatinine concentration range of 0–50 μg/mL to simulate patient sample conditions on the platform. Absorbance levels were measured in the last microreservoir. As the amount of creatinine in the serum increased, the signal level decreased as expected, and a statistical difference was observed in FBS solution for concentration levels ≥20 μg/mL (i.e., ≥2 mg/dL) compared to the control group (0 μg/mL), as shown in Figure 4c. Creatinine was also measured using the Jaffe method as the gold standard method (Figure S12). It was observed the detection signal of the Jaffe method was also correlated with the creatinine concentration. Moreover, the HSV value was measured with a creatinine concentration range of 0–50 μg/mL, and there is a statistical difference ≥20 μg/mL compared to the control group likewise absorbance measurements in FBS (Figures 4d and S11). The creatinine measurements in FBS showed lower signal levels than the measurements in PBS signal levels because the presence of different molecules and proteins in FBS may have affected the capture of creatinine on the electromechanical platform. Signal levels were saturated for ≥30 μg/mL creatinine in FBS (Figure S13). The platform allowed automated analysis of creatinine using 180 μL in PBS or 18 μL FBS in ∼50 min down to ∼1 and ∼2 mg/dL in PBS and FBS, respectively. The plug-and-play feature of the electromechanical LOC platform allows the creatinine to be detected without the need for technical personnel and bulky instruments. Color measurement, which determines the amount of creatinine, is made from a phone camera reducing the platform cost. With the use of the platform, critical changes in creatinine levels could be detected, and the necessary intervention could be made rapidly for the patient.

### Storage Conditions

**Figure 5:**
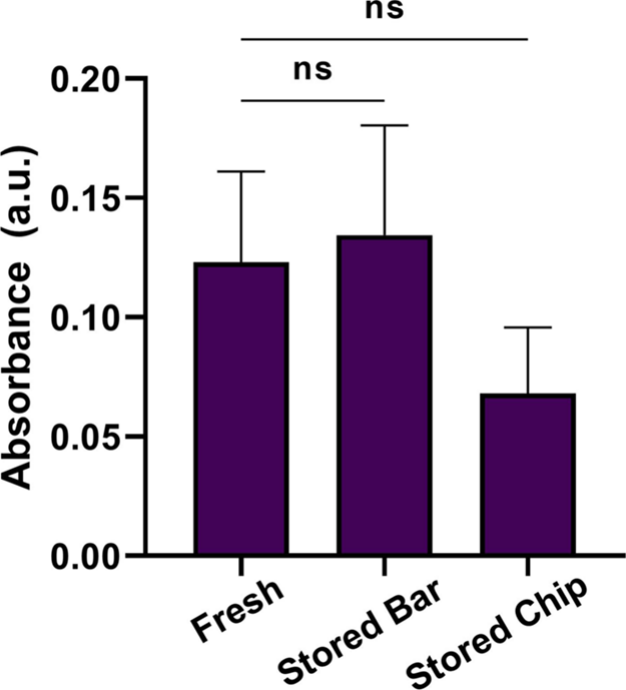
Comparison of the detection signal obtained from the freshly prepared chip and stirring bar, stirring bar stored at +4 °C for 6 months, and microreservoir chip stored at −20 °C for 2 months. For the experiments, 5 μg/mL creatinine prepared in PBS was used. ns indicates a nonsignificant *p*-value (*p* > 0.05).

We investigated the long-term stability of the stirring bar and microreservoir chip filled with assay solutions to assess the usability of the detection method in clinical or home settings. For this purpose, stirring bars were stored at −20 and + 4 °C for 6 months. We observed that the cover glasses were detached from the stirring bars stored at −20 °C. On the other hand, the stirring bar stored at +4 °C showed a similar performance compared to the freshly prepared bars (Figure 5). The microreservoir chip was maintained at −20 and + 4 °C for 2 months. The solutions were evaporated, and color change was observed in microreservoirs stored at +4 °C. However, these problems were not seen for the chip stored at −20 °C. Although the detection signal was slightly decreased for stored chips, no statistical signal difference was observed considering freshly prepared chips (Figure 5). These examinations show the potential of the developed system to be used in point-of-care settings when the required storage conditions are provided.

## Conclusions

We showed a new electromechanical LOC platform to detect creatinine automatically. The electromechanical system on the platform provided the movement and mixing of the stirring bars on different microreservoirs filled with assay solutions. Colorimetric detection of creatinine was conducted on the platform using a smartphone camera. The obtained detection signal, the mean intensity values of HSV spaces, showed a similar signal profile obtained with a benchtop spectrophotometer. The cost of the portable electromechanical LOC platform is ∼$90, and the cost for creatinine testing is ∼$2.7 per analysis (Table S1). These costs could be further reduced with mass production. The platform allows the detection of creatinine down to 1 and 2 mg/dL in PBS and FBS, respectively, which can be used to monitor CKD patients. Automated detection is offered with this platform in ∼50 min using only a minute amount of the serum sample (18 μL). Consumables of the platform (i.e., stirring bar and microreservoir chip filled with assay solutions) can be stored for long-term usage. Hence, the end-user could use this platform with a plug-and-play approach without the need for an expert in point-of-care settings with its portable and inexpensive design. The electromechanical LOC platform eliminates the need for pumps or valves or spectrometers that can increase the cost of detection systems. Therefore, compared to previously reported LOC/biosensor-based creatinine detection systems, the proposed electromechanical LOC platform allows more cost-effective and automated analysis (Table S2). Although the device could sense elevated creatinine levels in serum, the signal level and linear operation range can be improved further. For this purpose, the surface area of the stirring bar could be enlarged with nanomaterials to capture more analyte efficiently.^41−43^ The platform could be modified with different antibodies and assay solutions further to detect different biomarkers for different disease monitoring. In this way, the platform could become a generic detection platform that could democratize and disseminate disease-screening tests.

## Supporting Information Available

Figures S1–S13 and Tables S1 and S2. Materials; solutions; fabrication of chips containing microreservoirs; fabrication of stirring bars; mixing performance of electromechanical lab-on-a-chip platform; electromechanical lab-on-a-chip platform; surface functionalization of the glass substrate; creatinine detection; platform cost; and comparison (PDF)
Movie 1. Automated control of the electromechanical lab-on-a-chip platform (MP4)
