# Supplementary material for: An Electromechanical Lab-on-a-Chip Platform for Colorimetric Detection of Serum Creatinine
Source: ACS Omega. 2022 Jul 15;7(29):25837–43. doi: 10.1021/acsomega.2c03354 (PMC9330075; doi:10.1021/acsomega.2c03354)
Supplement: Supplementary file 1 — ao2c03354_si_001.pdf [file ao2c03354_si_001.pdf]

AN ELECTROMECHANICAL LAB-ON-A-CHIP PLATFORM FOR COLORIMETRIC DETECTION OF SERUM CREATININE

SUPPORTING INFORMATION

Betul Karakuzu[1], Ergun Alperay Tarim[1], Cemre Oksuz[1], and H. Cumhur Tekin[1, 2*]

[1]Department of Bioengineering, Izmir Institute of Technology, Izmir 35430, Turkey

[2]METU MEMS Center, Ankara 06520, Turkey

*Corresponding author e-mail: cumhurtekin@iyte.edu.tr

Table of Contents

Materials……………………………………………………………………………S3

Solutions……………………………………………………………………………S3

Fabrication of chips containing microreservoirs……………………………………S3

Fabrication of stirring bars……………………………………………………………S4

Figure S1 | In-house developed cuvettes……………………………………………S5

The mixing performance of the electromechanical lab-on-a-chip platform ………S5

Electromechanical lab-on-a-chip platform …………………………………………S6

Figure S2 | The electromechanical platform circuit for automatically conducting creatine detection protocol ……………………………………………………………S6

Figure S3 | Voltage depended rotational speed of the stirring bar connected to the DC motor ………..S6

Figure S4. An Android-based mobile application has been developed to control creatinine detection protocols remotely ……………………………………………S7

Figure S5 | Automated movement of the stirring bar in a sequence……………S8

Movie 1 | Automated control of the electromechanical lab-on-a-chip platform……S8

Surface functionalization of the glass substrate……………………………………S8

Figure S6 | FTIR results after 3-MPS, GMBS, Protein G, and anti-creatinine antibody incubations on the glass surface ……………………………………………S9

Figure S7 | Optimization of Protein-G concentration ……………………………S10

Figure S8 | Optimization of anti-creatinine antibody concentration………………S10

Figure S9 | Absorbance measurements of surface-functionalized glass coverslips with different concentrations of anti-creatinine antibody (10 and 100 µg/mL) at a creatinine concentration of 0 µg/mL……S11

Creatinine detection ……S11

Figure S10 | Mean intensity values of a) red, b) green and c) blue channels of captured images for different creatinine concentrations prepared in PBS……S11

Figure S11 | Masked images of microreservoirs in HSV color space for different creatinine experiments conducted in a) PBS and b) FBS samples……S11

Figure S12 | Detection of creatinine concentrations in FBS by Jaffe method……S12

Figure S13 | Absorbance values for creatinine detection in FBS……S12

Platform cost ……S13

Table S1 | Cost of the platform and single creatinine test ……S13

Comparison ……S14

Table S2 | Comparison of lab-on-a-chip systems/biosensors for creatinine detection……S14

References ……S14

Materials

(3-Mercaptopropyl) trimethoxysilane (3-MPS) (175617), bovine serum albumin (BSA), dialyzed FBS (F0392), and Pluronic F-127 were purchased from Sigma Aldrich (Missouri, USA). 200 proof Ethanol (K50690883 844) was purchased from Merck (Darmstadt, Germany). Goat anti-mouse IgG (H+L) with Alexa Flour™ Plus 488, PBS, N-γ-maleimidobutyryl-oxysuccinimide ester (GMBS), and Protein-G were acquired from Thermo Fisher Scientific (Massachusetts, USA). Anti-creatinine sheep polyclonal antibody (ab30719), creatinine (ab143309), and 3,3’,5,5’-tetramethylbenzidine (TMB) ELISA substrate (ab171522) were obtained from Abcam Inc (Cambridge, UK). Horseradish peroxidase (HRP)-creatinine conjugate (80-1133) was bought from Fitzgerald (Massachusetts, USA). 76×26 mm cover glasses were purchased from Marienfeld (Lauda-Königshofen, Germany). Round cover glasses (CS-3R) with 3 mm diameter were obtained from Harvard Apparatus (Massachusetts, USA). Poly(methyl methacrylate) (PMMA) plates with a thickness of 3 mm were purchased from Ata Pleksi (Istanbul, Turkey). The clear photoreactive resin (V2 FLGPCL04) was taken from FormLabs (Massachusetts, USA). Double side adhesive (DSA) (OCA8146-3) was acquired from Thorlabs (New Jersey, USA). Food dye was purchased from Ozmen Food Products (Izmir, Turkey).

Solutions

Distilled water with a resistance value of 0.5 MΩ was prepared using Sartorius Arium® (Sartorius, Goettingen, Germany). 70% Ethanol solution was made by diluting 70% (v/v) 200 proof Ethanol in distilled water. 3-MPS solution was obtained by diluting 4% (v/v) 3-MPS in 200 proof Ethanol. GMBS solution was made by diluting 1.2 g/mL GMBS in PBS. Pluronic solutions were prepared by diluting Pluronic F-127 (v/v) in PBS. Protein-G and anti-creatinine antibody solutions were prepared in PBS. HRP-creatinine conjugate solution was made in 0.5% Pluronic solution. Different concentrations of creatinine samples were prepared in PBS and also FBS. The food dye solution was obtained by diluting 1% (v/v) food dye in distilled water.

Fabrication of chips containing microreservoirs

A microreservoir chip was composed of PMMA pieces and a glass slide (76×26 mm). The chip was designed with 5 microreservoirs to contain specific assay solutions. Each microreservoir had a cylindrical shape of 8 mm in diameter and 6 mm in height. To fabricate these microreservoirs, 3 mm thick PMMA and DSA were cut with a laser cutter (Epilog Zing 16, Epilog Laser, Colorado, USA). After PMMA pieces were cleaned in 70% Ethanol, two layers of PMMA pieces were bonded together with

DSA, and then they were fixed to a glass slide, which was cleaned in an ultrasonic bath with 70% ethanol for 10 min, again with DSA.

Fabrication of stirring bars

A stirring bar containing creatinine-specific antibodies was composed of a PMMA bar and 3 mm diameter cover glass placed on the bar's tip. The PMMA bar was fabricated by cutting the 3 mm thick PMMA plate with a laser cutter to a width of 3 mm and a length of 23.5 mm. Then, this bar was cleaned with 70% Ethanol for 5 min. Cover glasses were also processed to be covered with creatinine-specific antibodies. For this purpose, cover glasses were cleaned in an ultrasonic bath with 70% Ethanol for 10 min and dried with nitrogen gas. Then, 100 W air plasma at 0.5 mbar (Zepto Plasma System, Diener, Ebhausen, Germany) was applied to these glasses for 2 min to activate their surfaces. To conduct the surface chemistry protocols on these tiny glasses, glasses were transferred to in-house developed cuvettes (Figure S1). Afterward, glasses were incubated with 3-MPS for 45 min at room temperature in the dark. After glasses were washed with ethanol, they were dried at room temperature. Later, glasses were incubated with GMBS for 45 min at room temperature in the dark, Protein-G overnight at +4°C, and anti-creatinine antibody at +4°C for 30 min, respectively. After each incubation step, glasses were washed with PBS. The glass functionalized with creatinine-specific antibody was placed on the tip of the PMMA bar, where 3D printer resin was covered and exposed to UV light for 10 s to be bonded [1]. Thus, the stirring bar was obtained.

To check surface chemistry success, the glass substrates were analyzed using Fourier transform infrared spectroscopy (Spectrum Two, Perkin Elmer, Massachusetts, USA). After Protein-G and anti-creatinine antibody binding steps, the glass substrates were treated with 50 µg/mL of Alexa Flour™ Plus 488-labeled goat anti-mouse IgG (H+L) antibody to analyze the efficiencies of each critical step. After the treatment, the surface was washed with 0.1% Pluronic solution and visualized on an inverted fluorescence microscope (Zeiss Axio Vert A1, ZEISS, Jena, Germany).

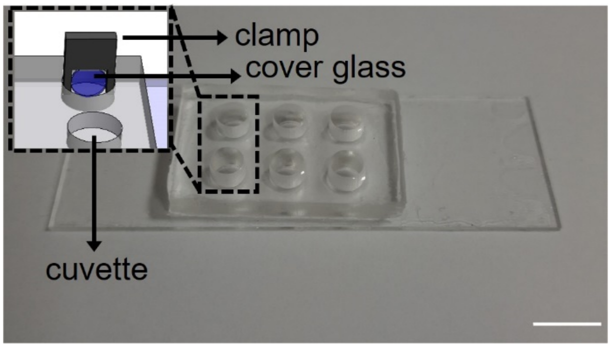

Figure S1. In-house developed cuvettes. Cuvettes were fabricated from PDMS with molding. Each cuvette has a volume of 60 µL, and glass slides were placed inside these cuvettes with a 3D-printed PLA clamp to be removed easily from the cuvettes without damaging their surfaces. The scale bar is 10 cm.

The mixing performance of the electromechanical lab-on-a-chip platform

A rapid mixing can be achieved by rotating the stirring bar inside microreservoirs. To evaluate the mixing performance, 1 µL blue color dye solution was pipetted in a microreservoir filled with 99 µL PBS. With/without rotating a stirring bar inside this microreservoir, color homogeneity appeared in the microreservoir with time was analyzed from the microreservoir's micrographs captured using an inverted microscope. The mixing index was used to measure the color homogeneity so the mixing efficiency on the micrographs, as follows [2]:

$$\mathrm{MI}\,(t) = \sqrt{\frac{1}{N}\sum_{k=1}^{N}\left(\frac{I(t,k)-I_{avg}(t)}{I_{avg}(t)}\right)^2} \qquad (1)$$

, where MI(t) is the mixing index at time t, N represents the total number of pixels, $I(t,k)$ shows the intensity of k pixels at time t, and $I_{avg}(t)$ is the average intensity value of the pixels at time t.

Electromechanical lab-on-a-chip platform

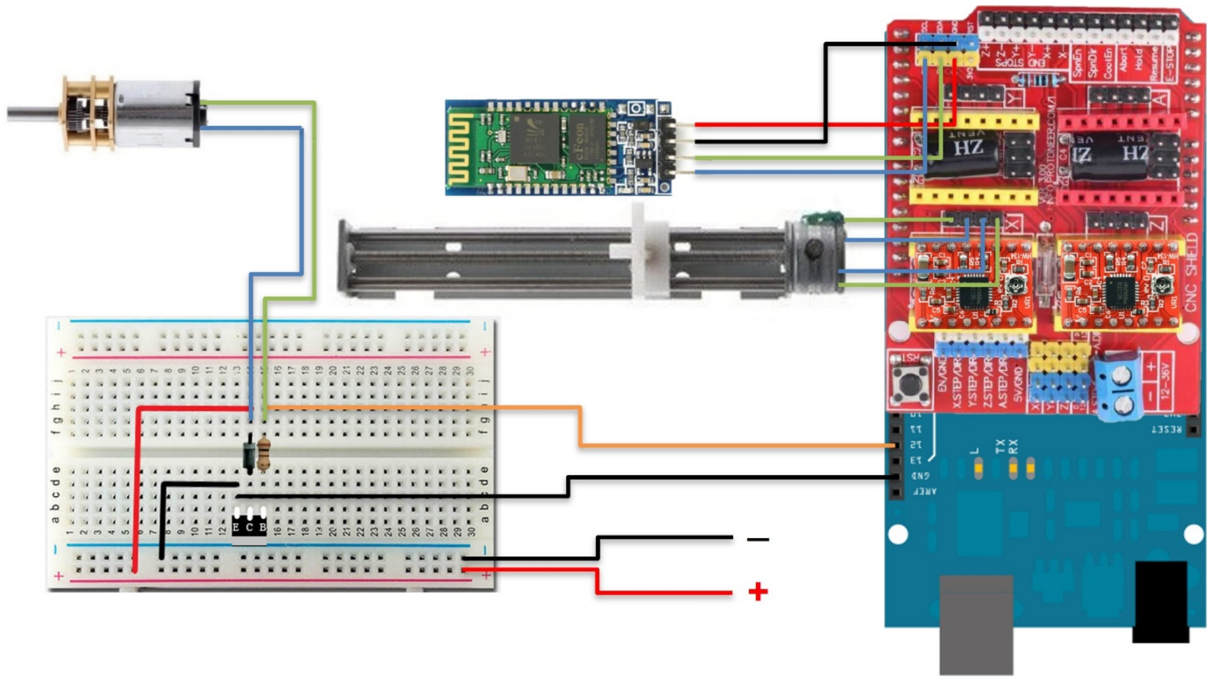

Figure S2. The electromechanical platform circuit for automatically conducting creatine detection protocol. The platform has two DC stepper motors for 2-dimensional movement, a DC motor for mixing the solutions inside microreservoir, and a Bluetooth module for remote control of the platform. All elements are controlled with the Arduino microprocessor equipped with a motor shield.

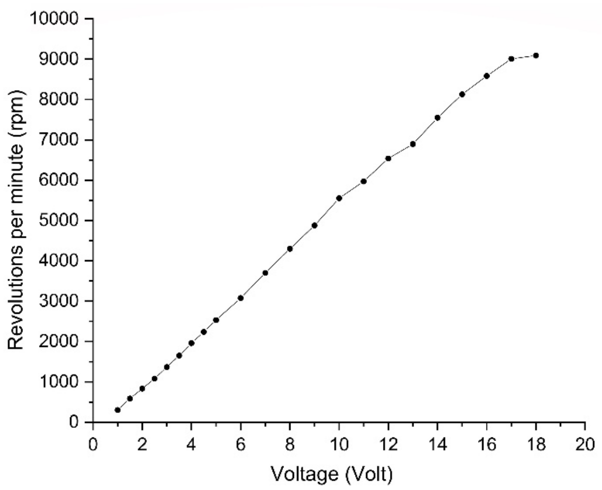

Figure S3. Voltage depended rotational speed of the stirring bar connected to the DC motor.

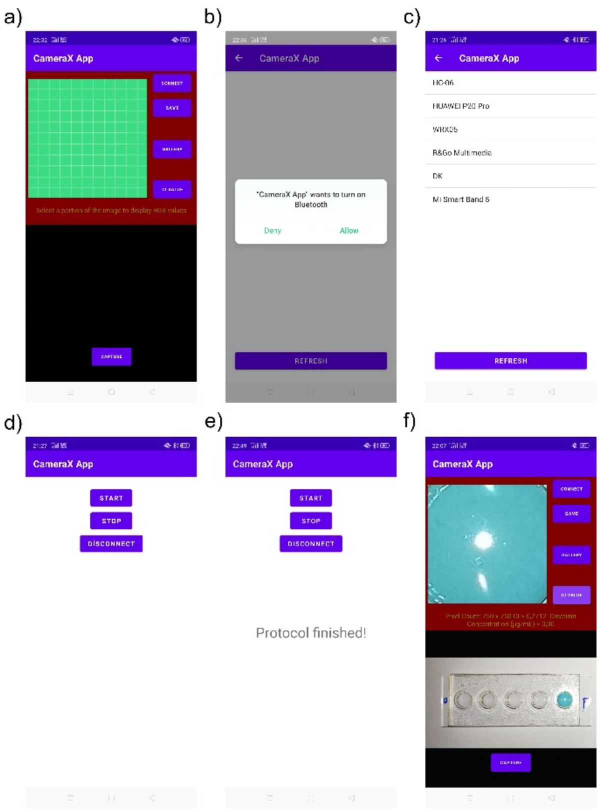

Figure S4. An Android-based mobile application has been developed to control creatinine detection protocols remotely. The software interface was designed in the Android Studio environment. (a) The mobile application opens with the user interface that reflects the camera image to the screen. (b) The Connect button is used for connection to the Bluetooth devices and when this button is pressed, the application warns the user to turn on the Bluetooth of the mobile device. (c) When Bluetooth is turned on, nearby and registered Bluetooth devices are displayed. By selecting the HC-06 Bluetooth model of the platform, (d) the buttons for starting, stopping and disconnecting the protocol appear. (e) "Protocol finished!" notification is coming when the creatinine detection protocol is finished. (f) Afterward, the image obtained as a result of the protocol on the application can be cropped and saved for analysis.

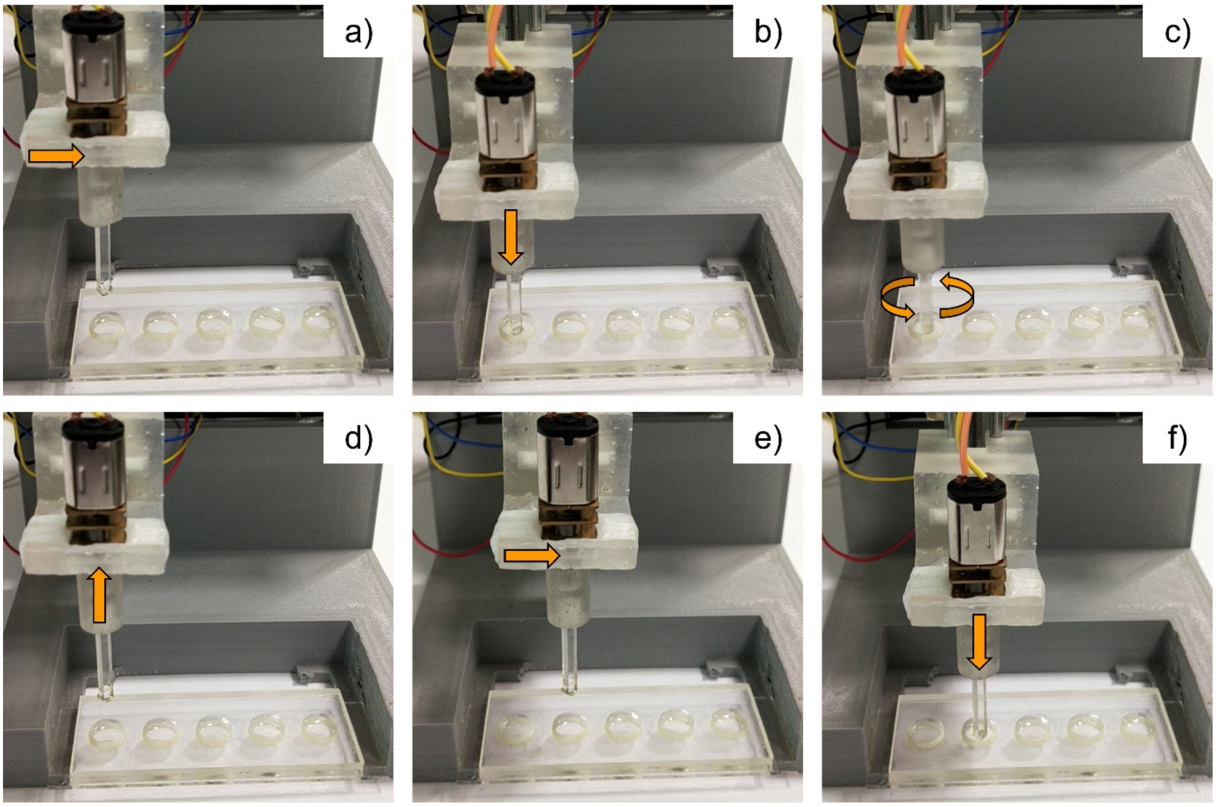

Figure S5. Automated movement of the stirring bar in a sequence. The stirring bar can a) go on top of a microreservoir, b) enter and c) rotate inside it, d) exit from it, and e) move and f) enter to the next microreservoir.

Movie 1. Automated control of the electromechanical lab-on-a-chip platform.

Surface functionalization of the glass substrate

The stirring bar contained 3 mm diameter cover glass on its tip to capture creatinine specifically. The success of the surface functionalization protocols was evaluated with FTIR analysis (Figure S6). 3-MPS has Si-OR and SH bonds in its molecular structure. The peaks of these bonds were observed at 960, and 2250 cm^{-1} absorption values, respectively [3]. In the molecular structure of GMBS, there are 3° amine, C=C in ring, C=O, and alkane bonds, and the peaks of these bonds were observed at 1110-1250, 1560, 1720-1820, and 2850-2950 cm^{-1} absorption values [3,4]. In the molecular structure of Protein-G, there are 1° amine, C-O, C=O, alkane, and OH bonds, and the peaks of these bonds were observed at absorption values of 770/3390, 1280, 1720, 2960, and 3300 cm^{-1}, respectively [3]. Similar peaks were also observed after anti-creatinine antibody immobilization. But, the magnitudes of peaks for OH group (3300 cm^{-1}) and 1° amine (3390 cm^{-1}) gradually altered with Protein-G and antibody absorptions. As a result, it was concluded that the glass surface was successfully functionalized with anti-creatinine antibodies.

The concentration of Protein-G solution used for surface functionalization protocols was also studied to determine the concentration to saturate the glass surface. As Protein-G concentration increased up to 100 µg/mL, the amount of fluorescent IgG bound on the glass surface increased (Figure S7). After this concentration, the amount of the fluorescent signal, and so the IgG bound on the surface did not change. Therefore, 100 µg/mL Protein-G was chosen to be used in the functionalization protocols. Similarly, analyzes were performed to determine the concentration of the anti-creatinine antibody. As the surface becomes saturated with anti-creatinine antibodies, it is expected that less fluorescent IgG will be captured on the surface, and the fluorescent signal on the surface will decrease. The fluorescent signal on the surface decreases as the anti-creatinine antibody concentration increases up to 100 µg/mL (Figure S8). After this concentration, the fluorescent signal remains stable. Moreover, the effect of antibody concentration on creatinine detection was examined. Equal absorbance signals were observed for 10 µg/mL and 100 µg/mL antibodies while detecting 0 µg/mL of creatinine (Figure S9). Because of that, 10 µg/mL creatinine antibody was used in the platform.

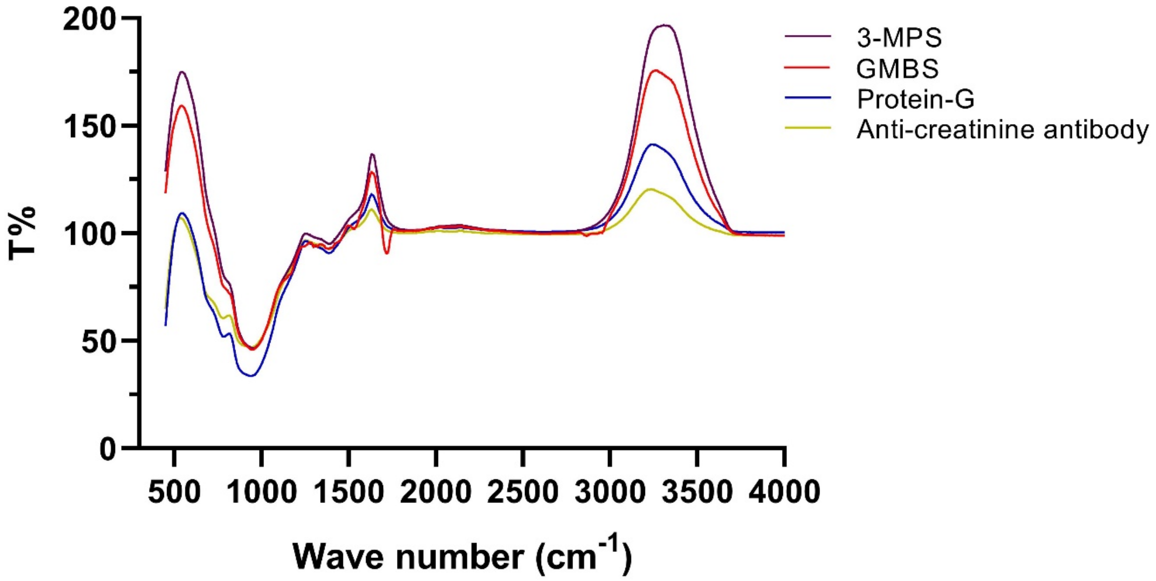

Figure S6. FTIR results after 3-MPS, GMBS, Protein G, and anti-creatinine antibody incubations on the glass surface.

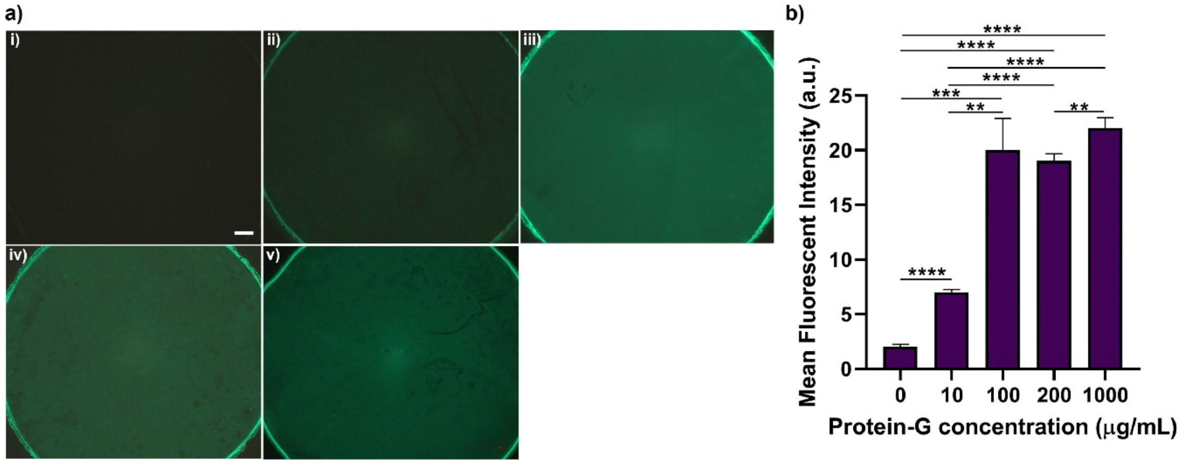

Figure S7. Optimization of Protein-G concentration. a) Fluorescence microscopy images of glass surfaces under a 5× objective. The applied Protein-G concentrations were i) 0 µg/mL ii) 10 µg/mL, iii) 100 µg/mL, iv) 200 µg/mL and v) 1000 µg/mL. The scale bar is 200 µm. b) Mean fluorescent intensities on the glass surface incubated with different Protein-G concentrations after fluorescent IgG treatment. Statistical differences between groups were analyzed by one-tail t-test. (**), (***) and (****) indicates $p<0.01$, $p<0.001$ and $p<0.0001$, respectively.

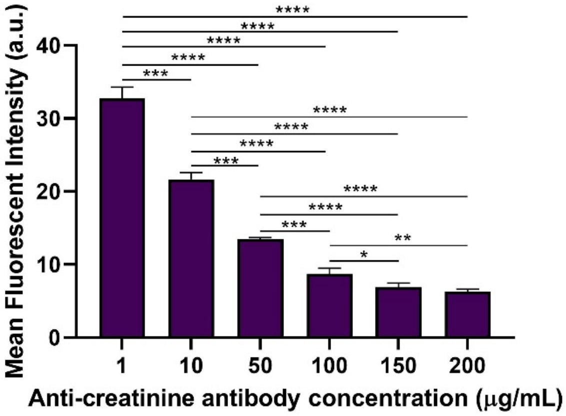

Figure S8. Optimization of anti-creatinine antibody concentration. Mean fluorescent intensities on the glass surface incubated with different anti-creatinine antibody concentrations after fluorescent IgG treatment were shown. Statistical differences between groups were analyzed by one-tail t-test. (*), (**), (***) and (****) indicates $p<0.05$, $p<0.01$, $p<0.001$ and $p<0.0001$, respectively.

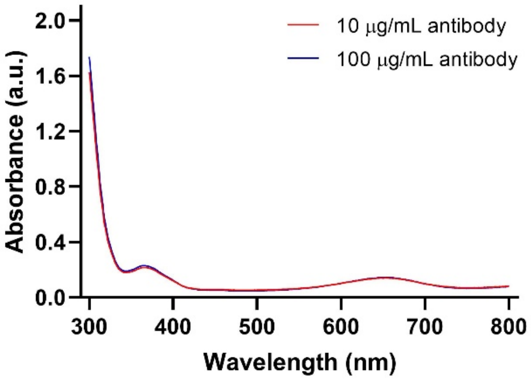

Figure S9. Absorbance measurements of surface-functionalized glass coverslips with different concentrations of anti-creatinine antibody (10 and 100 µg/mL) at a creatinine concentration of 0 µg/mL. In this experiment, TMB incubation was conducted for 10 min with 1000 rpm stirring.

Creatinine detection

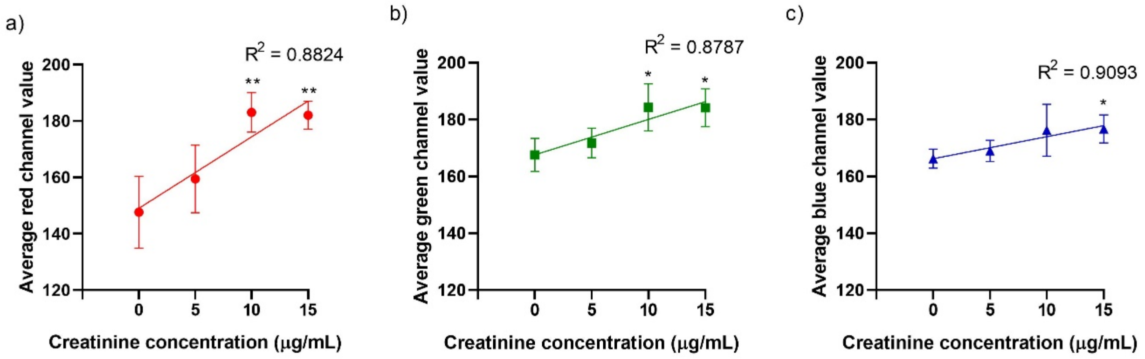

Figure S10. Mean intensity values of a) red, b) green and c) blue channels of captured images for different creatinine concentrations prepared in PBS. Statistics were performed by unpaired t-test and comparing each experimental group with 0 µg/mL as a control group. (*) and (**) indicates $p<0.05$ and $p<0.01$, respectively.

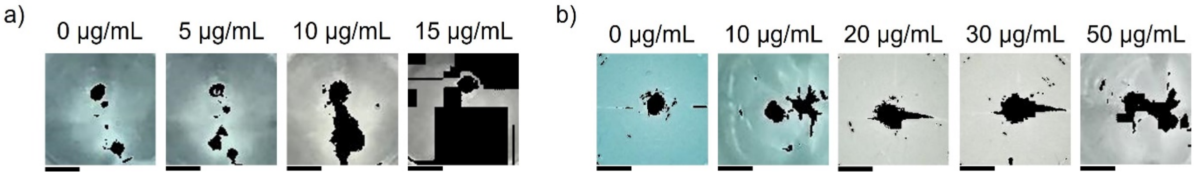

Figure S11. Masked images of microreservoirs in HSV color space for different creatinine experiments conducted in a) PBS and b) FBS samples. The scale bars are 2 mm.

Verification of creatinine in the samples: The FBS-creatinine solution was also analyzed on the microplate using the Jaffe method (Figure S12). For this, 20 µL of 1% Picric acid solution (Isolab, Germany), 20 µL of 3% NaOH (S8045, Sigma-Aldrich, USA), 30 µL of ultrapure water, and 50 µL of samples were added to the microplate for each concentration value [5]. Samples with creatinine were prepared in FBS and diluted at 1:10 (v:v) with PBS. Then, the microplate was incubated at 100 rpm on a shaker (NB-T205, N-Biotek, South Korea) at 27°C for 15 min. As a result of the reaction, absorbance measurement is taken at 520 nm using a spectrometer.

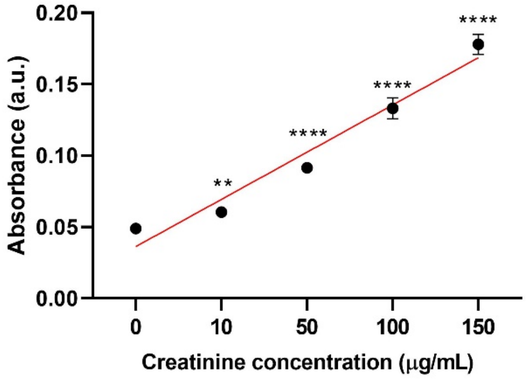

Figure S12. Detection of creatinine spiked in FBS by Jaffe method. R^2 value is 0.9539. Statistical differences according to 0 µg/mL creatinine measurements were analyzed by one-tail t-test. (**), and (****) indicates p<0.01, and p<0.0001, respectively.

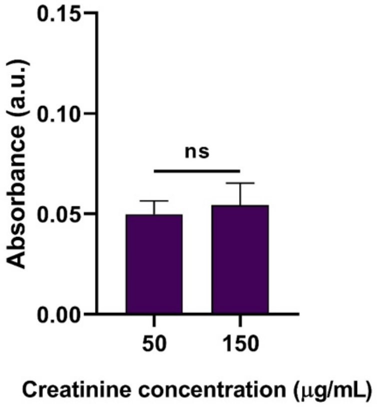

Figure S13. Absorbance values for different creatinine concentrations spiked in FBS. ns indicates non-significant *p*-value (*p*>0.05).

Platform cost

Table S1. Cost of the platform and single creatinine test.

	**Component**	**Cost**
**Electromechanical Platform**	Arduino Mega 2560 Rev3	$43.02
	Stepper Motor	$10.8 × 2
	DC Motor	$15.57
	PLA Filament	$4.29 (200 g)
	A4988 stepper motor driver	$1.35 × 2
	CNC Expansion Board	$2.39
		**Total: $89.57**
Consumables for Single Test		
**Microreservoir Chip**	PMMA	$0.072
	Glass Slide	$0.0712
	Double-sided Adhesive	$0.2060
	HRP-creatinine conjugate	$0.086
	TMB	$0.11
	PBS	$0,13168
		**Total: $0.68**
**Stirring Bar**	3-MPS	$0.12
	GMBS	$0.27
	Protein-G	$0.26
	Antibody	$0.037
	Round Glass	$1.16
	Resin	$0.11
		**Total: $1.96**

Comparison

Table S2. Comparison of lab-on-a-chip systems/biosensors for creatinine detection.

**References**	[6]	[7]	[8]	[9]	[10]	[11]	[12]	[13]	This study
**Detection time (min)**	<5	60	0.025	3	40	6	15	5	~50
**Sample volume (μL)**	40	2	NR	300	60	5	50	5	18
**Limit of detection (mg/dL)**	0.46	NR	~0.0023	NR	NR	0.19	0.11	0.76	~2
**Detection method**	Electro-chemical	Electrical	Electro-chemical	Electro-chemical	Electro-chemical	Optical	Optical	Optical	Optical
**Sample type**	Blood	Reference sample	Human urine	Serum	Reference sample	Blood	Urine	Plasma	Serum
**Automated analysis**	No	No	No	No	Yes	Yes	No	Yes	Yes
**Cost per test ($)**	NR	NR	NR	NR	NR	NR	NR	5	~2.7
**Cost of device ($)**	NR	NR	NR	NR	NR	NR	NR	300	~90
*NR: Not Reported.									

References

(1) Kecili, S.; Tekin, H. C. Adhesive Bonding Strategies to Fabricate High-Strength and Transparent 3D Printed Microfluidic Device. *Biomicrofluidics* **2020**, *14* (2), 024113.

(2) Tekin, H. C.; Sivagnanam, V.; Ciftlik, A. T.; Sayah, A.; Vandevyver, C.; Gijs, M. A. M. Chaotic Mixing Using Source–Sink Microfluidic Flows in a PDMS Chip. *Microfluidics and Nanofluidics* **2011**, *10* (4), 749–759.

(3) Nadeau, J. L. *Introduction to Experimental Biophysics: Biological Methods for Physical Scientists*; CRC Press, **2017**.

(4) The LibreTexts. *Spectroscopy of Amines*, **2022**.

(5) Toora, B. D.; Rajagopal, G. Measurement of Creatinine by Jaffe's Reaction-Determination of Concentration of Sodium Hydroxide Required for Maximum Color Development in Standard, Urine and Protein Free Filtrate of Serum. *Indian J Exp Biol* **2002**, 40 (3), 352-354.

(6) Wei, F.; Cheng, S.; Korin, Y.; Reed, E. F.; Gjertson, D.; Ho, C.; Gritsch, H. A.; Veale, J. Serum Creatinine Detection by a Conducting-Polymer-Based Electrochemical Sensor to Identify Allograft Dysfunction. *Anal Chem* **2012**, *84* (18), 7933–7937.

(7) Guha, S.; Warsinke, A.; Tientcheu, C. M.; Schmalz, K.; Meliani, C.; Wenger, C. Label Free Sensing of Creatinine Using a 6 GHz CMOS Near-Field Dielectric Immunosensor. *Analyst* **2015**, *140* (9), 3019–3027.

(8) Fekry, A. M.; Abdel-Gawad, S. A.; Tammam, R. H.; Zayed, M. A. An Electrochemical Sensor for Creatinine Based on Carbon Nanotubes/Folic Acid/Silver Nanoparticles Modified Electrode. *Measurement* **2020**, *163*, 107958.

(9) Dasgupta, P.; Kumar, V.; Krishnaswamy, P. R.; Bhat, N. Serum Creatinine Electrochemical Biosensor on Printed Electrodes Using Monoenzymatic Pathway to 1-Methylhydantoin Detection. *ACS Omega* **2020**, *5* (35), 22459–22464.

(10) Yasukawa, T.; Kiba, Y.; Mizutani, F. A Dual Electrochemical Sensor Based on a Test-Strip Assay for the Quantitative Determination of Albumin and Creatinine. *Analytical Sciences* **2015**, *31* (7), 583–589.

(11) Tseng, C.-C.; Yang, R.-J.; Ju, W.-J.; Fu, L.-M. Microfluidic Paper-Based Platform for Whole Blood Creatinine Detection. *Chemical engineering journal* **2018**, *348*, 117–124.

(12) Nakamoto, K.; Kurita, R.; Sekioka, N.; Niwa, O. Simultaneous On-Chip Surface Plasmon Resonance Measurement of Disease Marker Protein and Small Metabolite Combined with Immuno-and Enzymatic Reactions. *Chem Lett* **2008**, *37* (7), 698–699.

(13) Dal Dosso, F.; Decrop, D.; Pérez-Ruiz, E.; Daems, D.; Agten, H.; Al-Ghezi, O.; Bollen, O.; Breukers, J.; de Rop, F.; Katsafadou, M. Creasensor: SIMPLE Technology for Creatinine Detection in Plasma. *Anal Chim Acta* **2018**, *1000*, 191–198.
